# Current status of the training and work for emergency specialist nurses: A cross-sectional study in eastern China

**DOI:** 10.1097/MD.0000000000048329

**Published:** 2026-04-17

**Authors:** Chunyan Li, Qianqian Hu, Juan Yuan, Jingjing He, Yixin Wang, Sijing Peng

**Affiliations:** a School of Nursing, Anhui University of Chinese Medicine, Hefei, Anhui Province, China; b Key Laboratory of Geriatric Nursing and Health, Anhui University of Chinese Medicine, Hefei, Anhui Province, China; c Nanjing Drum Tower Hospital, The Affiliated Hospital of Nanjing University Medical School, Nanjing, Jiangsu Province, China; d School of Social and Behavioral Sciences, Nanjing University, Nanjing, Jiangsu Province, China.

**Keywords:** emergency, specialist nurse, status, train, work

## Abstract

Emergency specialist nurses play a critical role in improving the quality of emergency care and enhancing patient satisfaction. In China, there is a growing need to systematically evaluate the training and deployment of these nurses to better inform educational and managerial policymaking. This cross-sectional study, conducted in Jiangsu Province between December 2021 and February 2022, sought to comprehensively investigate the training experiences and professional status of emergency specialist nurses. Specific objectives included: assessing perceptions of the training program, including curriculum design, faculty composition, and instructional methods; identifying post-training developments in core competencies and career trajectories; and exploring key challenges and support mechanisms in their current practice. A convenience sample of 567 certified nurses completed a structured questionnaire. Key findings revealed that 63.8% of participants called for the inclusion of experienced clinicians in the training faculty, while 72.5% identified nursing research and teaching as the most needed areas for reinforcement. Only 48.5% regarded online teaching as effective. Training coordination was primarily managed by head nurses (71.4%). Although participants reported substantial improvements in clinical, teaching, managerial, and research capabilities after training, 19.2% had moved to nonemergency departments. The study concludes that while the existing training framework is structurally sound, several areas require attention: online training formats need optimization, the quality of clinical teaching should be enhanced, and greater emphasis should be placed on scientific research training. Continuous professional development also remains essential. A key limitation of this study is its reliance on convenience sampling and self-reported data without instrument validation.

## 1. Introduction

With the advancement of society and the evolution of the modern medical model, the role of emergency medicine has gained increasing significance. Emergency patients often present with rapidly changing conditions and a broad spectrum of diseases, which necessitates that emergency nurses possess multidisciplinary theoretical knowledge and practical skills to deliver comprehensive, specialized, and high-quality nursing care. The National Nursing Development Plan (2021–2025) in China emphasizes the training of specialist nurses in high-demand areas such as emergency care, intensive care, and pediatrics.^[1]^ Therefore, it is imperative to strengthen the training of emergency specialist nurses to enhance the quality of emergency care and patient satisfaction.

The United States has the longest history of cultivating specialist nurses. According to the American Academy of Nurse Practitioners, an Advanced Practice Nurse is defined as a licensed nurse with specialized clinical expertise and advanced practice proficiency. The American Academy of Nurse Practitioner also specifies that Advanced Practice Nurses must complete specialized training, obtain certification from a relevant professional body, and hold a master’s degree or higher.^[2]^ In 1976, the University of Virginia School of Nursing launched the first emergency nursing specialist program, followed by the University of Texas Health Science Center at Houston, which introduced a master’s program in emergency nursing in 1994. In the U.S., emergency specialist nursing is categorized into 3 main tracks: adult, pediatric, and neonatal emergency care. The curriculum encompasses foundational emergency nursing knowledge as well as specialized training in areas such as toxicology, trauma, and injury management.^[3]^ Additionally, students are required to complete advanced courses in pathophysiology, pharmacology, and health assessment, along with a minimum of 500 hours of clinical practice.^[4]^ Graduates are eligible to sit for the specialty certification examination.^[5]^

In the United Kingdom, emergency nurse practitioners emerged in response to clinical needs, with specialist practice services first introduced in 1986. In 1992, the Royal College of Nursing established criteria for emergency nurse practitioners, stating that they must be competent in comprehensive assessment, physical diagnosis, treatment prescribing, and health promotion, and must hold a master’s degree.^[6]^

In China, the concept of specialist nurses was introduced in the late 1980s to early 1990s, with formal training programs commencing in the 21st century. Emergency specialist nurses are defined as registered nurses with substantial expertise and clinical experience in emergency care who have obtained formal certification. Emergency nursing was 1 of the first 5 specialties selected for pilot training programs promoted by the Ministry of Health and has since been implemented nationwide.^[7]^ Since 2002, regions such as Beijing, Zhejiang, and Hunan have initiated emergency specialist nurse training. However, training standards in mainland China have yet to be unified. Trainees are typically required to hold a bachelor’s degree or higher and have prior emergency care experience. Most programs are short-term, lasting 2 to 3 months, and cover emergency skills, theoretical knowledge, nursing management, research methods, and communication skills. Training usually combines theoretical instruction with clinical mentorship, while assessments include written tests, practical examinations, objective structured clinical examinations, and thematic presentations.^[8,9]^

To improve the quality of emergency specialist nurse training, it is essential to evaluate current training content, delivery methods, assessment approaches, and post-certification professional activities in clinical practice, education, management, and research. Therefore, this study conducted a questionnaire survey among emergency specialist nurses in Jiangsu Province to investigate the current state of training and professional practice, with the aim of providing insights for optimizing the emergency specialist nurse training system.

## 2. Methods

### 2.1. Study setting and duration

Emergency specialist nurse training bases recruited eligible trainees and provided theoretical and practical training. The theoretical component covered topics including domestic and international status and recent advances in emergency medicine and critical care, emergency nursing management, humanistic care in emergency settings, as well as nursing research and education. The practical component focused on fundamental emergency rescue techniques, advanced life support, emergency nursing monitoring, assistance with invasive examinations or treatments, and the operation and management of common emergency equipment. The program, which typically lasted 1 to 3 months, was accredited by the Jiangsu Provincial Nursing Association or respective Municipal Nursing Associations. Trainees underwent assessment through theoretical tests, practical skill evaluations, or comprehensive examinations. Upon successful completion, the Nursing Association awarded them the Emergency Specialist Nurse Qualification Certificate.

This cross-sectional study employed a convenience sampling method to survey emergency specialist nurses in Jiangsu Province between December 2021 and February 2022. Inclusion criteria were: possession of an emergency specialist nurse certificate issued by the Chinese Nursing Association or a provincial-level authority, and voluntary participation in the study. Exclusion criteria were: no longer employed in a hospital setting, and inability to complete the questionnaire fully for any reason. The sample size was determined based on 5 to 10 times the number of questionnaire items.^[10]^ With 31 items (excluding general information) and considering an potential invalid response rate of 15%, the required sample size ranged from 183 to 379. The study was approved by the Ethics Committee of Nanjing Drum Tower Hospital (Approval No. 2022-013-02), and all participants provided informed consent voluntarily.

### 2.2. Survey tools

The questionnaire was developed by 3 researchers through a comprehensive literature review,^[8,11]^ with final items determined after 2 expert panel meetings. It consisted of the following sections: general information: including gender, age, professional title, education level, position, years of working experience, years in the emergency department, current work unit, level of involvement in the emergency specialist nursing program, and time since certification. Training feedback: comprising 25 items across 5 domains: curriculum, faculty, training quality characteristics, training management, and personal recommendations. Work status of specialist nurses: including 6 items on current work areas and perceived improvement in specialized clinical practice, teaching, management, and scientific research (File S1, Supplemental Digital Content, https://links.lww.com/MD/R689). The questionnaire utilized a mix of single-choice, multiple-choice, and fill-in-the-blank questions, with the personal recommendations section being open-ended.

### 2.3. Data collection

As trainees had returned to their respective hospitals, face-to-face questionnaire distribution was unfeasible. Data were collected electronically using the Questionnaire Star platform. The principal investigator contacted the directors of provincial emergency specialist nurse training bases, explaining the study purpose and questionnaire completion guidelines. Training base directors then distributed the questionnaire link via WeChat groups of graduated trainees. To ensure data accuracy and validity, the platform was set to allow only one submission per device, and all questions (except for personal suggestions) were mandatory. After collection, all responses were double-checked, and invalid questionnaires (e.g., those with excessively short completion times, contradictory answers, or uniform responses across all items) were excluded.

### 2.4. Data analysis

Data from the online survey platform were exported and analyzed using SPSS 26.0 (IBM Corp., Armonk). Categorical data were summarized as frequencies and percentages. Continuous data were presented as means and standard deviations.

## 3. Results

### 3.1. General information of emergency specialist nurses

A total of 575 questionnaires were collected from approximately 700 emergency specialist nurses surveyed in Jiangsu Province. After excluding 8 invalid responses, 567 valid questionnaires were retained, yielding a valid response rate of 98.61%. The participants were from 13 cities in the province, with the largest numbers from Nanjing (148), followed by Zhenjiang (67), Wuxi (66), Nantong (59), and Suzhou (53). The majority of respondents were female (93.47%). The median age was 37 years (33–41). Most held bachelor’s degrees (97.18%) and worked in tertiary hospitals (77.60%). The median years of total work experience were 15 (11–20), with 11 years (8–15) spent in the emergency department. The median time since specialty certification was 5 years (3–8). Of the trainees, 61.90% participated in provincial-level training programs, while the remainder attended municipal-level programs. Additionally, 84.13% completed both theoretical and practical training at the same site. Detailed demographic characteristics are presented in Table 1, and the distribution of work units is provided in Table 2.

**Table 1:** General information of emergency specialist nurses.

Category	Number (percentage, %)	Category	Number (percentage, %)	Category	Number (percentage, %)
*Gender*		*Title*		*Work years*	
Female	530 (93.47)	Professor of nursing	15 (2.65)	4~	69 (12.17)
male	37 (6.53)	Associate professor of nursing	167 (29.45)	10~	330 (58.20)
*Age*		Nurse-in-charge	314 (55.40)	20~	146 (25.75)
25~	26 (4.59)	Nurse	71 (12.52)	30~	22 (3.88)
30~	349 (61.55)	*Position*		*Work years in emergency department*	
40~	176 (31.04)	chief nursing officer and above	34 (6.00)	0~	209 (36.86)
50~	16 (2.82)	Head nurse/associate head nurse	220 (38.80)	10~	293 (51.68)
*Education level*		Nursing team leader	165 (29.10)	20~	62 (10.93)
Master and above	13 (2.29)	Other	5 (0.88)	30~	3 (0.53)
Bachelor	551 (97.18)	None	143 (25.22)	*Length of time already certified as a specialist nurse (yr*)	
College degree and below	3 (0.53)	*Training programme level*		<1	22 (3.88)
*Level of work hospital*		Provincial	351 (61.90)	1~	246 (43.49)
Tertiary A	309 (54.50)	Municipal	216 (38.10)	5~	216 (38.10)
Tertiary B	131 (23.10)	*Whether theoretical and practical training are at the same base*		10~	83 (14.64)
Secondary A	94 (16.58)				
Secondary B	30 (5.29)	Yes	477 (84.13)		
First-level	3 (0.53)	No	90 (15.87)		

**Table 2:** Work department of emergency specialist nurse (n = 567).

Department	Number (percentage, %)
Rescue room, prehospital care	274 (48.32%)
EICU, ICU	21 (3.70%)
Consultation room, observation room, infusion room, emergency ward, triage room	38 (6.70%)
Fever clinic	5 (0.88%)
Emergency room (regional rotations)	140 (24.69%)
Medical and surgical wards	45 (7.94%)
Other	44 (7.76%)

EICU = emergency intensive care unit, ICU = intensive care unit.

### 3.2. Training feedback from emergency specialist nurses

Regarding curriculum design, 30.51% of specialist nurses considered the balance between theoretical and practical components to be appropriate but indicated that research-related content was insufficient. A majority (72.49%) reported that nursing research and teaching were the most needed areas for enhancement, followed by quality control and management in emergency nursing (70.55%), new emergency specialty techniques (52.73%), and psychological adjustment for nurses (51.50%). Some respondents also suggested incorporating content related to nursing humanities, communication, and laws or regulations. In terms of instructional interaction, 43.49% reported that 21% to 40% of course time involved interactive activities, while only 0.53% indicated almost no interaction.

Concerning faculty composition, 14.81% indicated that clinical instructors primarily held intermediate or senior titles but lacked involvement of senior experts. The instructor-to-student ratio was mostly 1:2 (57.14%), followed by 1:1 (27.79%). Regarding the desired composition of the training team, 63.84% preferred the inclusion of experienced clinicians, followed by clinical nursing experts (61.02%), senior specialist nurses (47.09%), and professional teachers from medical schools (33.51%).

Most respondents (95.06%) reported having a dedicated instructor throughout the training who provided guidance in theory, practice, teaching, and research. Instructor training methods primarily included clinical teaching (78.29%), micro-lectures (71.61%), thematic discussions (65.31%), telephone communication (46.75%), and email (33.21%). Only 7.76% considered the number of complex cases in the training base to be low.

In terms of training management, 71.43% reported that overall coordination was primarily handled by head nurses, followed by trained specialist nurses (24.87%). Some bases were coordinated by base directors, nursing department staff, or training program directors. Nearly all respondents (98.58%) indicated that training bases had strict management systems that were consistently implemented.

Regarding training formats, 91.89% found workshops most beneficial, followed by clinical practice (90.83%), scenario simulation (85.54%), offline theoretical instruction (76.72%), and nursing rounds (71.08%). Online teaching was considered least effective (48.50%). For assessment methods, 81.13% considered research design most helpful, followed by objective structured clinical examination (75.84%), hands-on skill assessment (74.07%), and theoretical examination (62.26%). Some trainees also suggested group examinations as an effective approach.

In open-ended suggestions, some nurses noted that clinical instructors had limited teaching time due to heavy workloads and recommended increasing instructor numbers and reasonably arranging teaching schedules. Others highlighted a lack of nursing research training and suggested expanding related content. Several participants proposed that training bases organize regular continuing education and feedback opportunities, such as inviting experienced specialist nurses for exchange sessions, to support professional development after certification.

### 3.3. Current work status of emergency specialist nurses

Among the 567 respondents, 109 (19.22%) had transferred out of the emergency department to other units such as medical/surgical departments, administrative offices, or equipment management. Participants were divided into 2 groups based on current work status: those retained in the emergency department and those who had left. Significant differences were observed between the groups in sex, age, professional title, total work experience, training level, and years since specialty certification (all *P* < .05). Details are provided in Table 3.

**Table 3:** Analysis of influencing factors associated with emergency specialist nurses’ retention in the emergency department.

Factor	Category	Total (n = 567)	Still working in ED (n = 458)	Left ED (n = 109)	Test statistic	*P*-value
Sex	Male	37	37	0	χ^2^ = 9.42	.002
	Female	530	421	109		
Hospital grade	Tertiary A	309	249	60	χ^2^ = 7.608	.107
	Tertiary B	131	102	29		
	Secondary A	94	82	12		
	Secondary B	30	24	6		
	Primary	3	1	2		
Age (yr)	< 30	26	24	2	χ^2^ = 26.947	<.001
	30–45	488	405	83		
	> 45	53	29	24		
Professional title	Senior	15	10	5	χ^2^ = 23.706	<.001
	Associate senior	167	117	50		
	Intermediate	314	265	49		
	Junior	71	66	5		
Education level	Master’s or above	13	10	3	χ^2^ = 0.159	.859
	Bachelor’s	551	445	106		
	College or below	3	3	0		
Total work experience (yr)	>10	452	351	101	χ^2^ = 13.981	<.001
	≤10	115	107	8		
ED work experience (yr)	>10	284	238	46	χ^2^ = 3.357	.067
	≤10	283	220	63		
Training level	Provincial	351	267	84	χ^2^ = 13.15	<.001
	Municipal	216	191	25		
Years since specialist certification	>5	232	158	74	χ^2^ = 40.61	<.001
	≤5	335	300	35		

ED = emergency department.

Regarding specialty competence enhancement, 64.37% of certified specialist nurses had participated in lectures or practical skill competitions and received awards, followed by participation in academic activities and further training (55.56%), serving as nursing team leaders (51.68%), implementing specialty nursing programs (47.44%), applying new nursing techniques (43.92%), participating in nursing consultations (40.39%), applying for utility or invention patents (33.51%), and pursuing further academic education (2.29%). Some also reported roles as leaders of hospital emergency nursing teams.

In terms of management skills, 92.42% had participated in quality improvement projects or quality control circle activities, and 36.33% had been promoted to head nurse or higher positions. For teaching capacity, 88.10% had conducted or participated in nursing teaching activities within the emergency department or hospital-wide, 79.54% had taught nursing students or new nurses, 32.98% had participated in college or university teaching, and 29.10% had become specialist nurse mentors. Some also served as American Heart Association instructors or leaders of emergency specialist nurse training bases.

Regarding research output, 54.67% had published articles in domestic non-SCI/SSCI journals, 32.80% in domestic statistical source journals, and only 2.47% in international journals. In terms of research projects, 19.93%, 8.99%, 1.23%, and 0.18% had undertaken hospital-level, municipal-level, provincial-level, and national-level scientific research projects, respectively.

## 4. Discussion

### 4.1. Suboptimal effectiveness of online training

Survey results indicated that 98.58% of specialist nurses acknowledged the presence of a strict management system at training bases, which was conscientiously implemented, with dedicated personnel coordinating the entire training process. With the rapid advancement of technology, online education has gained an important role in medical education due to its advantages of spatiotemporal flexibility, relatively low cost, and diverse formats.^[12,13]^ In China, some specialist nurse training programs have incorporated online theoretical teaching, yet its effectiveness requires further evaluation.^[14,15]^ This study found that online theoretical teaching was perceived as the least helpful training format among respondents. Potential reasons may include unstable network platforms, susceptibility of trainees to environmental distractions or viewing fatigue, and low learner engagement, all of which may adversely affect the learning experience and outcomes. Therefore, more effective modes of online teaching require further exploration. For instance, Xu Nengmeng^[16]^ developed a three-layer virtual platform for anesthesia nurse training (comprising practice, assessment, and data layers) that incorporated practice and self-test modes, utilized animation and modeling for theoretical and practical examinations, and employed professional support to ensure platform stability. This approach significantly improved trainees’ examination scores and self-directed learning ability. Liu Weiquan^[17]^ established a software platform featuring user account management, a resource library, training, examination, and competency evaluation modules, adopting a problem-discussion-guidance teaching method that enhanced both job competency and teaching satisfaction among specialist nurses. Similarly, Lin Jianhong^[18]^ implemented an online flipped classroom model where trainees were required to produce and upload preparatory videos, followed by in-class feedback and consolidation, which effectively enhanced learning motivation. Given the current state of online teaching for specialist nurses in China, training bases should seek to improve the digital literacy of instructors and administrators, equip programs with suitable remote classrooms and stable network platforms, and establish a feasible online education system to comprehensively ensure teaching quality.^[19]^

### 4.2. Shortcomings in clinical teaching

In open-ended feedback, some trainees reported that clinical instructors had limited time for teaching due to heavy clinical workloads, and that opportunities for hands-on practice were insufficient. Currently, most emergency specialist nurse training bases in China are located in tertiary general hospitals, where emergency nurses typically face high workloads and pressure. Balancing clinical duties with teaching responsibilities thus remains a challenge. First, greater emphasis should be placed on the selection of clinical instructors. Li Wei^[20]^ developed a core competency evaluation system for clinical instructors of specialist nurses through 2 rounds of expert consultation, highlighting the importance of clinical practice ability, teaching competence, research capability, professional development, organizational management, and critical thinking. Furthermore, targeted and standardized training for clinical instructors should be implemented, including the development of clear and feasible teaching plans and regular evaluation of teaching outcomes, to build a high-quality teaching team. This study revealed that the student-to-teacher ratio in clinical teaching was mainly 1:2 (57.14%), followed by 1:1 (27.79%), indicating a generally reasonable ratio. Base managers should determine trainee intake based on departmental nursing workload, teaching capacity, and educational demands. In addition, flexible scheduling and the involvement of experienced clinicians in teaching may help mitigate insufficient instructional support and ensure teaching quality.

### 4.3. Gaps in research-related knowledge and skills

In this study, 72.49% of specialist nurses indicated that nursing research content needed strengthening in training, and 81.13% considered research design an effective form of assessment. These findings reflect a strong demand for research knowledge among specialist nurses and a perceived insufficiency of existing research resources. Currently, nurses in China generally have relatively low academic qualifications and limited access to systematic research training, resulting in inadequate skills in scientific analysis and problem-solving. However, research output often influences career advancement and rewards, creating a mismatch between the supply and demand for nursing research training. Wu Qianyun^[21]^ in a study on clinical nurses’ research ability in Sichuan Province, reported that specialist nurses exhibited weaknesses in data processing and research design, suggesting that training in these areas should be prioritized. Yang Jian^[22]^ established tiered research objectives for nurses at different levels and enhanced the effectiveness of research training using a stratified-team-mentorship model. Other scholars have adopted approaches such as research salons,^[23]^ workshops,^[24]^ hands-on training,^[25]^ and restricted online courses^[26]^ to improve nurses’ research literacy in areas including topic selection, research design, data collection, statistical analysis, and manuscript writing. Training base administrators may consider increasing the proportion of nursing research content based on program duration and available resources, and adopt appropriate teaching methods to improve training quality and satisfaction.

### 4.4. Challenges in job role clarity and professional growth opportunities

This study found that 19.22% of emergency specialist nurses had transferred out of the emergency department to medical/surgical wards or administrative units. Statistical analysis indicated that sex, age, professional title, total work experience, training level, and time since certification were significantly associated with retention in emergency care roles. The high-stress and demanding nature of emergency care may contribute to these trends. Female nurses may experience reduced work engagement due to physical strain or family responsibilities, potentially leading to departure from the emergency setting. Older nurses and those with longer tenures may leave due to high burnout, lack of specialized career pathways, or the absence of well-defined training and utilization frameworks.^[27]^ Meanwhile, those with higher professional titles may transition into management roles in other departments. In terms of training-related factors, advanced training may raise specialist nurses’ professional expectations and autonomy, thereby expanding external career opportunities. The higher turnover among those certified longer may also reflect shortcomings in the current training system, particularly in providing continuous professional development and long-term incentive mechanisms.^[28]^

At present, specialist nurses in China still face challenges such as an emphasis on training over utilization and the lack of a scientific management system, which limits their potential professional impact. A qualitative synthesis^[29]^ of experiences of specialist nurses in China over the past decade revealed that respondents perceived ongoing insufficient resource support and suboptimal management models. Huang Daohua^[30]^ promoted the dissemination and outreach of nursing expertise by establishing specialist nurse clinics, organizing health poverty alleviation activities, public health education, and hosting specialist nursing months with trainee presentations to foster exchange and competition. Li Yuemei^[31]^ implemented performance management reforms through a special talent fund, effectively enhancing specialist nurses’ motivation and performance. Qin Hui^[32]^ developed and validated a work engagement scale tailored to specialist nurses within the Chinese cultural context to better assess their work status and improve specialist nursing services. In addition, some participants in this study expressed a desire for regular continuing education and post-training feedback opportunities. Currently, there are few domestic studies on retraining models for specialist nurses. Future research may draw on the training experiences of advanced practice nurses in other countries to explore feasible approaches to retraining formats, duration, content, and recertification within the Chinese context.

## 5. Limitations

This study has several limitations. First, the use of convenience sampling restricted participant recruitment to emergency specialist nurses within a single province in eastern China, which may limit the generalizability of the findings. Future multicenter studies with larger sample sizes are recommended to enhance the representativeness and broader applicability of the results. Second, the cross-sectional design and the absence of validated assessment scales constrained the depth of the investigation. Third, the data relied exclusively on self-reported responses, which may introduce subjective bias and limit interpretative rigor. Subsequent research would benefit from incorporating mixed-method approaches and psychometrically robust instruments to obtain a more comprehensive and objective understanding of the training and professional status of emergency specialist nurses.

## 6. Conclusion

This study examined the training characteristics and post-training professional activities of emergency specialist nurses in Jiangsu Province, China. The findings indicate that while training programs generally maintained rigorous management protocols, several areas require improvement. These include optimizing online teaching formats, enhancing the quality of clinical instruction, strengthening research-related training, and establishing structured continuing education mechanisms. Furthermore, there is a demonstrated need for hospital administrators to refine role delineation and career development pathways for specialist nurses to better leverage their expertise and support professional growth. Addressing these issues may contribute to more effective training outcomes and sustainable professional development for emergency specialist nurses in China.

## Author contributions

**Conceptualization:** Chunyan Li, Jingjing He.

**Data curation:** Chunyan Li, Jingjing He.

**Formal analysis:** Chunyan Li, Jingjing He, Sijing Peng.

**Investigation:** Chunyan Li.

**Methodology:** Chunyan Li, Qianqian Hu, Yixin Wang.

**Supervision:** Sijing Peng.

**Validation:** Qianqian Hu, Juan Yuan, Sijing Peng.

**Visualization:** Qianqian Hu, Sijing Peng.

**Writing – original draft:** Chunyan Li.

**Writing – review & editing:** Chunyan Li, Juan Yuan, Yixin Wang, Sijing Peng.

## Supplementary Material


