# Supplementary material for: Current status of the training and work for emergency specialist nurses: A cross-sectional study in eastern China
Source: Medicine (Baltimore). 2026 Apr 17;105(16):e48329. doi: 10.1097/MD.0000000000048329 (PMC13095306; doi:10.1097/MD.0000000000048329)
Supplement: Supplementary file 1 [file medi-105-e48329-s001.pdf]

# Supplemental file 1

## 【Chinese】

### 急诊急救专科护士培训基地学员反馈调查问卷

尊敬的临床护理老师：

您好！感谢您参加《急诊急救专科护士培训基地学员反馈调查问卷》。本次调查内容分为三部分，分别为专科护士一般资料、基地反馈调查和能力提升调查。本调查将占用您 20 分钟左右的时间，问卷将采用无记名的形式，所收集的资料仅用作护理研究，请您尽可能真实地填写个人信息。如您对问卷内容有任何疑问或不理解之处，欢迎随时咨询。

**第一部分：专科护士一般资料**

1.您的性别： ☐男 ☐女

2.您的单位等级：☐三甲 ☐三乙 ☐二甲 ☐二乙 ☐一级医院

3.您的年龄： （ ）岁

4.您的职称： ☐正高 ☐副高 ☐中级 ☐初级

5.您的学历： ☐硕士及以上 ☐本科 ☐专科及以下

6.您的职位： ☐科护士长 ☐护士长/副护士长 ☐护理组长 ☐其他（自填） ☐无

7.您的工作年限：（ ）年

8.您的急诊专科工作年限：（ ）年

9.您目前所在的工作区域（例：急诊科抢救室）：（ ）

10.您参与培训的基地医院名称：

11.您参与的专科护士培训级别：☐国家级（中华） ☐省级 ☐市级

12.您已取得专科护士资格证书（ ）年

**第二部分：培训基地学员反馈调查（课程、师资、质量特色、管理）**

1.培训课程是否系统前沿？

A.是，由不同模块组成，涵盖了急诊急救护理各个方面最新发展内容

B.系统但缺乏前沿性，部分知识未及时更新

B.前沿但缺乏系统性，包含的内容不够全面

C.一般，有点类似于学习班

2.培训课程各部分的比重安排如何？

A.理论较多，实践和科研较少

B.实践较多，理论和科研较少

C.理论和实践分配合理，科研较少

D.理论、实践及科研部分分配合理

3.培训课程的理论培训和临床实践衔接是否紧密？

A.很紧密，在某些理论课后立即安排相应临床实践以加深理解

B.较紧密，理论与实践有部分交叉延伸内容

C.不紧密，理论归理论，实践归实践

4.课程中安排互动的比例占整个培训课程的多少？

A.几乎没有 B.有，约占 0-20% C.有，约占 21%-40% D.有，约占 40%以上

5.培训中需要加强的内容有哪些？（多选）

☐.急救护理专科新技术 ☐急诊护理质控与管理 ☐护理科研与教学 ☐护士心理调适 ☐其他（自填）

6.理论教师的多学科合作

A.由医疗、护理及其他团队成员组成，比例合适

B.以专科医生为主，较少有专科护士参与

C.以专科护士为主，较少有专科医生参与

7.理论教师授课质量

A.大部分专家（三分之二或以上）备课认真，授课质量好

B.部分专家（一半左右）备课认真，授课质量好

C.大部分专家备课欠认真，质量一般

8.临床实践师资水平

A.有高水平专家参与带教，带教组由中高级职称老师组成，层次合理

B.带教组由中高级职称老师组成，但缺乏高水平专家

C.没有明确的带教组，缺乏系统的临床带教

9.临床实践老师的带教质量

A.非常好，理论、操作、科研相结合，给了我很大的帮助

B.比较好，主要在操作方面给予指导

C.一般，未能提供详细实用的临床教学

10.（如 1 名老师临床带教 2 名学员，即为 1:2）

11.您认为培训师资中还需要加入哪些？（多选）

A.临床护理专家　B.工作年限大于等于 15 年的专科护士 C.经验丰富的临床医生

D.医学院校教师

12.每位学员有固定的指导老师吗？

A.有　　　B.无（跳过下一题）

13.如果有，他/她通过什么方式对你指导？（可多选）

A.临床带教 B.专题讨论 C.邮件 D.微信 E.电话

14.基地是否存在病种不多，疑难病例少的情况？

A.是 B.否

15.基地是否存在护理人员不足，不能保证教学/带教质量的问题？

A.是  B.否

16.基地是否存在满足于知识灌输、技能培训、答辩过关训练，而对提高临床思维、综合解决问题能力的帮助不大的情况？

A.是 B.否

17.整个培训协调由谁专门负责？

A.护士长

B.经过培训的专职的（已不从事临床一线护理工作）专科护士

C.经过培训的专科护士，但仍然是临床一线护士

D.未经专科护士培训的护士或其他工作人员

E.无专人负责

18.培训基地负责人是否高度参与并主导整个培训？

A.除了上她的课，很少见到她/他

B.参与了培训工作，有时和我们在一起

C.基地负责人主导并高度参与培训工作，常和我们在一起

19.基地是否有严格的管理制度（例如请销假、上课打卡等）并认真执行？

A.有健全的管理制度并严格执行

B.有管理制度但执行不严

C.无管理制度

20.基地是否关心你的学习和生活？

A.非常关心，出台很多措施方便我们的工作和生活

B.比较关心，有时能为我们提供便利

C.不太关心

21.基地对你结业答辩的帮助

A.基地专门提供指导老师，主动辅导，有问必答

B.基地的老师提供一定的帮助

C.基地的老师没有提供帮助

22.您认为培训形式较有帮助的有哪些？（多选）

A.线下理论　B.线上理论　C.临床实践　D.护理查房

E.工作坊　F.情景模拟　G.其他（自填）

23.您认为考核形式较有帮助的有哪些？（多选）

A.理论考试　B.操作考试　C.OSCE 考核　D.科研设计答辩　E.其他（自填）

24.您对基地的总体满意度如何？

A.很满意　　B.满意　　C.一般　　D.不满意

25.您对整个专科护士培训过程有何宝贵建议？

**第三部分：培训基地学员能力提升情况调查（专科、管理、教学、科研）**

您目前是否仍在急诊科工作？☐是 ☐否

“否”跳转：于专科护士培训后第（　）年离开急诊科 现任部门为（　）

1.经培训后，您的专科能力获得了以下的提升（多选）

☐承担护理组长任务　☐参与护理会诊　☐参加讲课或技能比赛并获奖　☐参加学术活动、外出进修　☐开展专科护理特色项目　☐开展专科护理新技术　☐申请了实用专利或发明专利 ☐进一步学习获得了学历提升 ☐其他（自填）

2.经培训后，您的管理能力获得了以下的提升（多选）

☐ 参与质量改进项目、品管圈活动　☐升任护士长或更高职位　☐其他（自填）

3.经培训后，您的教学能力获得了以下的提升（多选）

☐承担科室护生、新护士带教活动 ☐参与科室及全院的护理查房、业务学习活动　☐成为专科护士导师　☐其他（自填）

4.经培训后，您的科研能力获得了以下的提升(多选）

☐在国内统计源期刊上发表论文（如有，请填篇数）

☐在国内非统计源期刊上发表论文（如有，请填篇数）

☐在国际学术期刊上发表论文（如有，请填篇数）

☐承担院级科研课题（如有，请填项目数）

☐承担市级科研课题（如有，请填项目数）

☐承担省部级科研课题（如有，请填项目数）

☐承担国家级科研课题（如有，请填项目数）

【English】

Questionnaire for Trainees of Emergency Specialist Nurse Training Centers

Dear Clinical Nursing Colleague,

Hello! Thank you for participating in the "Questionnaire for Trainees of Emergency Specialist Nurse Training Centers". This survey is divided into three parts: General Information of the Specialist Nurse, Training Center Feedback Survey, and Competency Improvement Survey. This survey will take approximately 20 minutes to complete. It is anonymous, and the collected data will be used solely for nursing research purposes. Please provide information as truthfully as possible. If you have any questions or find any part unclear, please feel free to ask.

Part 1: General Information of the Specialist Nurse

Gender: □ Male □ Female

Your Hospital Level: □ Tertiary A □ Tertiary B □ Secondary A □ Secondary B □ Primary Hospital

Your Age: ( ) years

Your Professional Title: □ Senior (Chief) □ Associate Senior □ Intermediate □ Junior

Your Education Level: □ Master's degree or above □ Bachelor's degree □ College diploma or below

Your Position: □ Department Head Nurse □ Nurse Manager / Associate Nurse Manager □ Team Leader □ Other (Please specify): ________ □ None

Your Total Years of Nursing Experience: ( ) years

Your Years of Experience in Emergency Specialty: ( ) years

Your Current Work Area/Unit (e.g., ER Resuscitation Room): ( )

Name of the Training Center Hospital You Attended:

Level of Specialist Nurse Training You Participated In: □ National □ Provincial □ Municipal

Years Since Obtaining Your Specialist Nurse Qualification Certificate: ( ) years

Part 2: Training Center Feedback Survey (Curriculum, Faculty, Quality Features, Management)

Was the training curriculum systematic and cutting-edge?

A. Yes, composed of different modules, covering the latest developments in all aspects of emergency nursing.

B. Systematic but lacking cutting-edge content; some knowledge was not updated timely.

C. Cutting-edge but lacking systematization; the content covered was not comprehensive enough.

D. Average, somewhat similar to a standard training course.

How was the distribution of weight among the different parts of the training curriculum?

A. More theory, less practice and research.

B. More practice, less theory and research.

C. Reasonable distribution between theory and practice, but less research.

D. Reasonable distribution among theory, practice, and research parts.

Was the connection between theoretical training and clinical practice in the curriculum tight?

A. Very tight, corresponding clinical practice was arranged immediately after certain theoretical sessions to deepen understanding.

B. Relatively tight, there was some overlap and extension between theory and practice.

C. Not tight; theory was theory, practice was practice.

What proportion of the entire training course was dedicated to interactive sessions?

A. Almost none

B. Yes, approximately 0-20%

C. Yes, approximately 21%-40%

D. Yes, approximately more than 40%

Which areas required strengthening in the training? (Select all that apply)

□ New technologies in emergency specialty nursing

□ Emergency nursing quality control and management

□ Nursing research and education

□ Psychological adjustment for nurses

□ Other (Please specify): ________

Multidisciplinary collaboration among theoretical instructors:

A. Composed of medical, nursing, and other team members, with an appropriate ratio.

B. Primarily composed of specialist physicians, with limited participation from specialist nurses.

C. Primarily composed of specialist nurses, with limited participation from specialist physicians.

Teaching quality of theoretical instructors:

A. Most experts (two-thirds or more) prepared seriously, and the teaching quality was good.

B. Some experts (about half) prepared seriously, and the teaching quality was good.

C. Most experts lacked serious preparation, quality was average.

Level of clinical practice instructors:

A. High-level experts participated in teaching; the teaching team consisted of intermediate and senior titles, with a reasonable hierarchy.

B. The teaching team consisted of intermediate and senior titles, but lacked high-level experts.

C. No clear teaching team, lacked systematic clinical teaching.

Teaching quality of clinical practice instructors:

A. Excellent, integrated theory, operation, and research, provided significant help.

B. Good, mainly provided guidance in operational aspects.

C. Average, failed to provide detailed and practical clinical teaching.

What was the student-to-teacher ratio during clinical practice? (e.g., 1 teacher for 2 students = 1:2) ( )

Which roles do you think should still be added to the training faculty? (Select all that apply)

A. Clinical Nursing Experts

B. Specialist nurses with ≥15 years of experience

C. Experienced clinical doctors

D. Medical school faculty

Did each trainee have a fixed supervisor/mentor?

A. Yes

B. No (Skip next question)

If yes, how did he/she provide guidance? (Select all that apply)

A. Clinical teaching

B. Seminars

C. Email

D. WeChat

E. Phone

Did the training center have insufficient case variety and a lack of complex cases?

A. Yes

B. No

Was there a shortage of nursing staff at the center that could not guarantee teaching/instruction quality?

A. Yes

B. No

Did the center tend to focus solely on knowledge transfer, skill training, and preparation for defense, offering little help in improving clinical thinking and comprehensive problem-solving abilities?

A. Yes

B. No

Who was specifically responsible for the overall coordination of the training?

A. Nurse Manager

B. A trained, full-time specialist nurse (no longer engaged in frontline clinical work)

C. A trained specialist nurse who remains a frontline clinical nurse

D. A nurse or other staff member without specialist nurse training

E. No one specifically responsible

Did the head of the training center participate highly and lead the entire training?

A. Apart from delivering their lectures, we rarely saw him/her.

B. Participated in the training work, sometimes spent time with us.

C. The center head led and highly participated in the training work, often spent time with us.

Did the center have strict management systems (e.g., for requesting leave, class check-in) and enforce them seriously?

A. Had sound management systems and strictly enforced them.

B. Had management systems but enforcement was lax.

C. Had no management systems.

Did the center care about your learning and life?

A. Very caring, implemented many measures to facilitate our work and life.

B. Relatively caring, sometimes provided conveniences for us.

C. Not very caring.

The center's assistance with your final project defense:

A. The center specifically provided guiding teachers, actively tutored, and answered all questions.

B. The center's teachers provided some help.

C. The center's teachers provided no help.

Which training formats did you find more helpful? (Select all that apply)

A. Offline theory

B. Online theory

C. Clinical practice

D. Nursing rounds

E. Workshops

F. Scenario simulation

G. Other (Please specify): ________

Which assessment formats did you find more helpful? (Select all that apply)

A. Theoretical exam

B. Practical / Operational exam

C. OSCE

D. Research project defense

E. Other (Please specify): ________

What is your overall satisfaction with the training center?

A. Very Satisfied

B. Satisfied

C. Neutral

D. Dissatisfied

What valuable suggestions do you have for the overall specialist nurse training process?

( )

Part 3: Competency Improvement Survey for Trainees (Specialty, Management, Education, Research)

Are you currently still working in the Emergency Department? ☐ Yes ☐ No

If "No": Left the Emergency Department ( ) year(s) after specialist nurse training. Current department is ( ).

After training, your specialist competencies have improved in the following areas (Select all that apply):

☐ Undertook Team Leader tasks

☐ Participated in nursing consultations

☐ Participated in lectures or skill competitions and won awards

☐ Participated in academic activities, external training/observerships

☐ Developed specialized nursing projects / characteristic services

☐ Implemented new specialist nursing technologies

☐ Applied for utility model or invention patents

☐ Pursued further education and obtained a higher academic degree

☐ Other (Please specify): ________

After training, your management competencies have improved in the following areas (Select all that apply):

□ Participated in quality improvement projects, Quality Control Circle activities

□ Promoted to Nurse Manager or higher position

□ Other (Please specify): ________

After training, your teaching competencies have improved in the following areas (Select all that apply):

□ Undertook mentoring activities for nursing students or new nurses in the department

□ Participated in departmental and hospital-wide nursing rounds, professional study sessions

□ Became a specialist nurse preceptor/mentor

□ Other (Please specify): ________

After training, your research competencies have improved in the following areas (Select all that apply, please indicate quantity if applicable):

□ Published papers in Chinese Statistical Source Journals (If yes, number: ___ )

□ Published papers in other Chinese journals (non-Statistical Source) (If yes, number: ___ )

□ Published papers in international academic journals (If yes, number: ___ )

□ Undertook hospital-level research projects (If yes, number: ___ )

□ Undertook municipal-level research projects (If yes, number: ___ )

□ Undertook provincial/ministerial-level research projects (If yes, number: ___ )

□ Undertook national-level research projects (If yes, number: ___ )
